# Effects of hepatitis B vaccination on hepatitis B surface antigen in neonates and its change in vivo

**DOI:** 10.2478/abm-2022-0029

**Published:** 2023-06-16

**Authors:** Shuqi Wang, Yuting Bai, Fangyuan Yuan, Ting Wang, Wenyi Luo, Can Luo, Qiang Wang, Dongsheng Wang

**Affiliations:** Department of Laboratory Medicine, Affiliated Hospital of North Sichuan Medical College, Nanchong, Sichuan 637000, China; North Sichuan Medical College, Nanchong, Sichuan 637000, China; Department of Clinical Laboratory, Sichuan Cancer Hospital and Institute, Sichuan Cancer Center, School of Medicine, University of Electronic Science and Technology of China, Sichuan 610041, China

**Keywords:** hepatitis B antibodies, hepatitis B surface antigens, hepatitis B vaccines, infant, newborn

## Abstract

**Background:**

Vaccination is effective to prevent hepatitis B virus (HBV) infection. However, there is still a risk of infection after vaccination. In clinical work, we found that newborns were positive for HBV surface antigen (HBsAg) after vaccination.

**Objectives:**

To determine the effect of hepatitis B vaccination on the detection of HBsAg trend in newborns.

**Methods:**

We collected data at birth, history of vaccination for hepatitis B, quantitative HBsAg results, and other information about newborns born in our hospital from July 2017 to July 2020. Serum samples from healthy neonates were randomly selected to be supplemented with recombinant hepatitis B vaccine on a concentration gradient, and HBsAg was measured quantitatively.

**Results:**

Data from 1417 neonates were included in the study; 306 (21.6%) were HBsAg positive within 8 d after vaccination, with levels ranging from 0.104 IU/mL to 0.339 IU/mL. The proportion of neonates with HBsAg-positive serum was significantly correlated with the level of hepatitis B surface antibodies (anti-HBs) in the serum of their mothers (*P* < 0.01). Experiments in vitro showed that the proportion of neonates with HBsAg-positive serum was correlated with the dose of the hepatitis B vaccine, and when the concentration of the hepatitis B vaccine reached 5 ng/mL and 10 ng/mL, the serum HBsAg levels showed a significant negative correlation with the original concentration of serum anti-HBs.

**Conclusions:**

Hepatitis B vaccination can affect the level of HBsAg detected in neonatal serum, and the effect could be mitigated by delaying the measurement. Moreover, maternal anti-HBs offset the effects of neonatal vaccination on HBsAg serum levels.

Hepatitis B virus (HBV) is a small hepatotropic enveloped virus composed of circular partial double-stranded DNA that replicates by reverse transcription in the intermediate form of RNA in hepatocytes [1]. HBV infection is a public health problem, especially in developing countries, and has been widely studied [2, 3]. HBV can cause acute hepatitis when it infects humans. The prognosis for acute infection is mostly related to age. After acute infection, approximately 95% of newborns, 20%–30% of children (aged £15 years), and <5% of adults will develop chronic infection [3, 4]. Infants and young children cannot effectively produce an adaptive immune response to clear HBV, and the immune system of the newborn is immature. Infection at birth or within 2 years after birth will usually result in chronic disease [5]. Therefore, identifying acute neonatal infections is important. Over time, chronic HBV infection without treatment can lead to liver fibrosis, cirrhosis, and hepatocellular carcinoma (HCC) [5]. According to a WHO report, the HBV infection rate was estimated to be 3.5% in 2015, and approximately 257 million people suffer from chronic hepatitis B (CHB) worldwide [2]. In China, the prevalence of HBV surface antigen (HBsAg) carriers declined from 9.8% in 1992 to 7.18% in 2006 because a nationwide hepatitis B immunization program for newborns was introduced in 1992 [6, 7]. A meta-analysis found that the prevalence of HBV infection in the general population of China from 2013 to 2017 was 6.89% [8]. Despite the declining prevalence of HBV, measures to achieve the WHO 2030 goal of eliminating hepatitis B on time still need improvement.

HBV is transmitted by exposure to blood or other specific body fluids (saliva, semen, or vaginal fluid) of an infected person, and transmission can occur from mother to child (MTCT) or from person to person [2, 9]. In China, MTCT transmission is the main route of transmission of hepatitis B, especially during pregnancies with high HBV loads [10, 11], and approximately 30%–50% of HBV-infected people acquire the virus through MTCT [12, 13]. Although the public’s concept of fertility has changed and China has introduced a new family planning policy, the domestic fertility rate has not risen as expected [14]. In fact, the proportion of older women has increased significantly, and the consequent risk of MTCT transmission in the perinatal period deserves our attention [15].

The WHO recommends that all newborns receive their first dose of hepatitis B vaccine within 24 h after birth [5]. HBsAg is the first viral marker in the serum of hepatitis B patients and can be used for early diagnosis and screening of hepatitis B [4]. HBsAg reappears as positive 6 months after the initial positive test, confirming the presence of CHB [5]. Low serum HBsAg may predict spontaneous or treatment-induced HBsAg seroclearance [16]. Hepatitis B surface antibodies (anti-HBs) are protective antibodies that mark recovery from acute infection or immunity from vaccination [4]. Generally, anti-HBs can be detected in patients with self-limiting hepatitis B infection only after HBsAg has disappeared from the blood. Both HBsAg and anti-HBs are positive in severe acute hepatitis, chronic active hepatitis, or different subtypes of superinfection.

Although newborns are vaccinated with hepatitis B vaccine alone or combined with hepatitis B immunoglobulin for standard active-passive immunoprophylaxis after birth, up to 9% of newborns are still infected with HBV through MTCT transmission [9, 17]. Currently, most of the hepatitis B vaccines used in China are recombinant hepatitis B vaccines. Vaccination with recombinant vaccine may lead to HBsAg-positive serum. In the early stage of applying the recombinant hepatitis B vaccine, researchers proposed that serum was HBsAg–positive because of the vaccination, known as hepatitis B surface antigenemia [18]. Subsequent studies have found that hepatitis B surface antigenemia can also occur in healthy blood donors and hemodialysis patients after hepatitis B vaccination and in adults in general [19,20,21].

These potential roles of HBsAg quantification apply to selected populations only [16]. How should the true significance of the results of HBsAg positivity in the quantitative detection of surface antigen after the newborn is injected with the vaccine be judged? Is the positivity caused by the recombinant hepatitis B vaccine? Or are newborns truly infected with HBV? Early and accurate judgment is of great importance for newborns, which, through antiviral therapy, can reduce the occurrence of CHB and subsequent damage to liver function caused by viral replication. In this study, by collecting the quantitative results of hepatitis B after neonatal injection of the recombinant hepatitis B vaccine, the duration of HBsAg positivity and the correlation between HBsAg positivity and the mother’s anti-HBs were explored, and the above problems were verified by randomly selecting the serum of healthy people to supplement with the recombinant hepatitis B vaccine. Because anti-HBs in the mother are mainly IgG immunoglobulin, which can be transmitted to the newborn via placental circulation, and the recombinant hepatitis B vaccine is mainly composed of HBsAg, after inoculation, the newborn may produce an antigen–antibody reaction, resulting in newborn anti-HBs after the injection of vaccine, creating an inevitable error regarding the amount of actual antibody. However, the mother’s anti-HBs level will not fluctuate significantly within a short period of time, and it can better reflect the actual level of newborn anti-HBs and is not affected by other factors. Therefore, in this study, the mother’s anti-HBs results were grouped to explore the relevant issues.

Currently, an effective measure to prevent and control HBV infection is timely and complete hepatitis B vaccination during the neonatal period. During clinical laboratory measurements, there are often cases of positive HBsAg or HBsAg and anti-HBs in the results of quantitative detection of hepatitis B in newborns, and tracing their mother’s medical history reveals no hepatitis B infection. To determine the effect of hepatitis B vaccination on the detection of HBsAg in newborns, the clinical data from 1,417 neonates hospitalized in the neonatology ward were analyzed and simulated.

## Methods

This study was approved by the Institutional Review Board at the Affiliated Hospital of North Sichuan Medical College (Certificate of approval no.2019ER(R)101-1).

### Patients

We conducted a retrospective analysis of data from newborns at the Affiliated Hospital of North Sichuan Medical College born from July 2017 to July 2020 using electronic medical records. The inclusion criteria were as follows: the patients were aged 1–30 d; hepatitis B vaccine (10 μg) was administered within 24 h after birth; hepatitis B immunoglobulin (200 μg) was administered at the same time if the mother was HBsAg positive, and hepatitis B markers were measured during hospitalization. The exclusion criteria were as follows: patients with multiple congenital hereditary and immunodeficiency diseases and those with admission causes such as premature delivery, neonatal jaundice, neonatal pneumonia, or septicemia. The following data were sought and recorded on clinical data forms: name, sex, birth weight, date and time of administration of hepatitis B vaccine, date and time of collection of blood specimens, and HBV infection history of the mother.

### Assays for HBsAg quantification

The HBsAg test used in this study was the Cobas HBsAg assay (Cobas E602, Roche). The results showed that HBsAg >0.06 IU/mL was positive, anti-HBs <10 mIU/mL was negative, 10 mIU/mL ≤ anti-HBs < 100 mIU/mL indicated a low level of antibodies, and anti-HBs ≥ 100 mIU/mL indicated a high level of antibodies.

### Serum verification experiment

The serum specimens of HBsAg (−)/anti-HBs (−) healthy subjects were numbered and an online random number generator was used to randomly select corresponding specimens to simulate newborns whose mothers were negative for anti-HBs. The sera of HBsAg (−)/anti-HBs (+) healthy subjects were chosen to simulate newborns whose mothers were positive for anti-HBs. Then, the recombinant hepatitis B vaccine (derived from Chinese Hamster Ovary cells) (North China Pharmaceutical Jintan Biotechnology) (20 μg/1.0 mL/dose) was added to the selected samples to adjust the concentration of the vaccine in the serum to 0.5 ng/mL, 1.0 ng/mL, 2.0 ng/mL, 5.0 ng/mL, and 10.0 ng/mL. Finally, we measured hepatitis B quantitatively.

### Statistical analysis

Statistical analyses were conducted using IBM SPSS Statistics for Windows, version 22.0. According to the normal test of the Q-Q chart, the positive results of HBsAg in newborns were distributed in a skewed way, and the mean value was represented by the median (quartile). A Fisher test was used to compare HBsAg results after vaccination of neonates born to antigen-negative and antigen-positive mothers. A chi-square test was used to compare the distribution of newborn HBsAg for mothers negative for surface antigen, but with different antibody concentrations. A rank sum test was used to compare the detection of HBsAg results in different serum groups after the recombinant hepatitis B vaccine was added to the serum. When the vaccine in serum was at 10 ng/mL, the linear correlation between HBsAg and anti-HBs results in the anti-HBs (+) group was determined by their Pearson correlation coefficient. *P* < 0.05 was considered significant.

## Results

### Analysis of quantitative detection results of hepatitis B in 1417 newborns

The 1417 neonates (783 male and 634 female) had been vaccinated with the hepatitis B vaccine within 24 h after birth, and among them, 306 (21.6%) were HBsAg positive. The average HBsAg-positive result was 0.186 IU/mL (range: 0.104–0.339 IU/mL). The neonatal HBsAg and anti-HBs results are shown in **Table 1**. In 306 cases of HBsAg-positive newborns, 66.7% of the neonates were negative for anti-HBs, 22.6% of the neonates had low levels of anti-HBs, and 10.8% of the neonates had high levels of anti-HBs.

**Table 1.** HBsAg and anti-HBs levels after 1417 newborns were vaccinated with the hepatitis B vaccine

**HBsAg**	**Number of newborns**	**Anti-HBs**		

		**<10 mIU/mL (%)**	**10–99 mIU/mL (%)**	**≥100 mIU/mL (%)**
≤ 0.06 IU/mL	1111	229 (20.6)	259 (23.3)	623 (56.1)
>0.06 IU/mL	306	204 (66.7)	69 (22.6)	33 (10.8)

Anti-HBs, hepatitis B surface antibodies; HBsAg, hepatitis B surface antigen.

**Figure 1.**
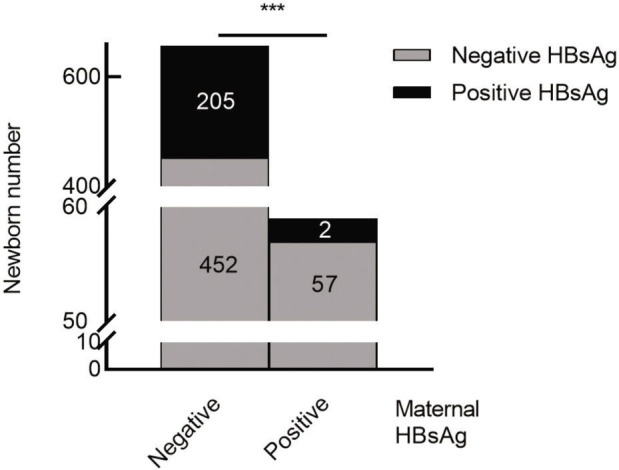
Newborn HBsAg status (black bars, positive; gray bars, negative) grouped by maternal HBsAg status. ***P < 0.01 (Fisher exact test). HBsAg, hepatitis B surface antigen.

Through electronic medical records and an examination report inquiry system, 716 maternal prenatal hepatitis B quantitative test results were collected, among which 59 mothers were HBsAg positive, accounting for 8.2%. The results of HBsAg analysis for newborns and mothers are shown in **Figure 1**. There were 205 (31.20%) cases with positive HBsAg in newborns born to mothers with negative HBsAg. By contrast, there were only 2 (3.4%) positive cases among the newborns whose mothers were positive for HBsAg. Because *P* < 0.001, we concluded that the distribution difference of HBsAg in neonates according to the mother’s HBsAg group is significant.

### HBsAg in newborns with different levels of anti-HBs in mothers and newborns

Neonatal cases with negative maternal HBsAg were selected (HBV immunoglobulin was not injected after birth) and were grouped according to the concentration of maternal anti-HBs. The results for neonatal HBsAg are shown in **Table 2**. When the HBsAg of the mother was negative, the level of positive HBsAg of the newborn was significantly inversely correlated with the increase in the concentration of anti-HBs of the mother (χ^2^ = 156.51, *P* < 0.001), indicating that the distribution of HBsAg detected after the newborn was inoculated in different maternal anti-HBs groups was significant. In the negative anti-HBs maternal group, 193 newborns without anti-HBs accounted for 85.8%; in the maternal group with a low level of anti-HBs, 133 newborns had a low level of anti-HBs, accounting for 79.5%; and in the maternal group with a high level of anti-HBs, 256 newborns had a high level of anti-HBs, accounting for 96.6%. This distribution trend of neonatal anti-HBs is consistent with that of maternal anti-HBs.

**Table 2.** HBsAg and the distribution of anti-HBs in neonates with different concentrations of anti-HBs in the mothers

**Newborn**	**Maternal anti-HBs (mIU/mL)**		

	**<10 (%)**	**10–99 (%)**	**≥100 (%)**
Number	225	167	265
HBsAg-positive rate (%)***	61.33	25.15	9.34
Anti-HBs <10 mIU/mL	193 (85.8)	8 (4.8)	4 (1.5)
10.00 IU/mL ≤ anti-HBs ≤ 99 IU/mL	24 (10.7)	133 (79.5)	5 (1.9)
Anti-HBs ≥ 100 mIU/mL	8 (3.6)	26 (15.7)	256 (96.6)

Anti-HBs, hepatitis B surface antibodies; HBsAg, hepatitis B surface antigen.

*** *P* < 0.01, comparing HBsAg-positive rate of neonatal serum with groups of different maternal anti-HBs levels.

**Figure 2.**
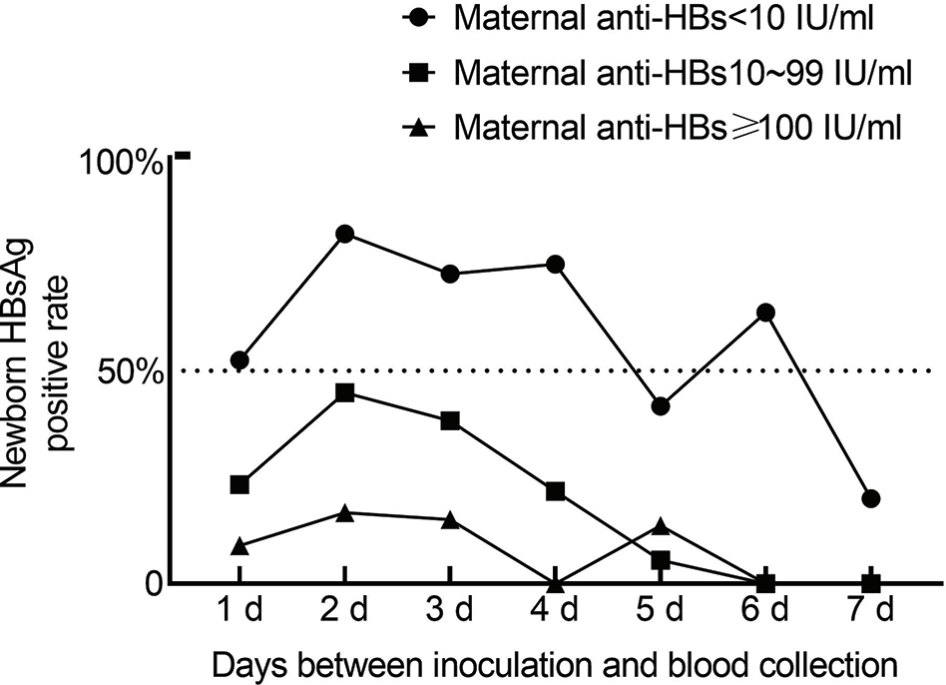
The proportion of neonatal HBsAg positives (mother with negative HBsAg) at various maternal anti-HBs concentrations (solid circles, <10 IU/mL; solid squares, 10–99 IU/mL; solid triangles, ≥100 IU/mL) and with days between vaccination and blood collection. Within 7 d of vaccination, among newborns with negative anti-HBs, the proportion of those (positive rate) of HBsAg was close to or >50%, while that for mothers with low and high levels of anti-HBs was <50%. Anti-HBs, hepatitis B surface antibodies; HBsAg, hepatitis B surface antigen.

The groups indicated above were further analyzed according to the days between vaccination and blood collection, and the results are shown in **Figure 2**. The figure shows that the overall trend of the positive rate of neonatal HBsAg increased 2 d before and then gradually decreased 2 d after vaccination, regardless of the maternal anti-HBs concentration. When the mother had no anti-HBs, the positive rate of HBsAg in the newborn was higher than that in mothers with normal and lower concentrations of anti-HBs.

### Metabolism of the hepatitis B vaccine in neonates

**Figure 3.**
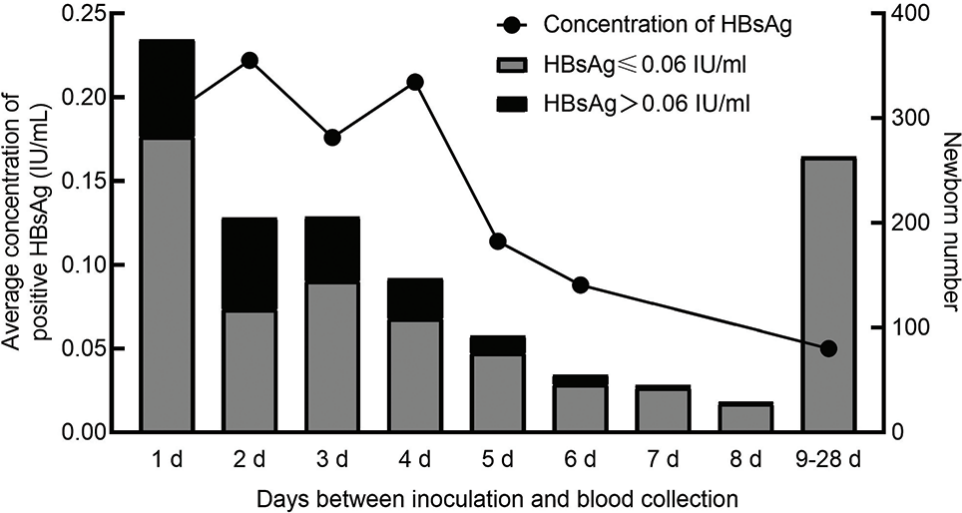
The proportion of neonates positive for HBsAg and the average concentration of HBsAg in neonates (gray ≤0.06 IU/mL; black bars >0.06 IU/mL) after vaccination (solid circles). Neonates continued to appear positive for HBsAg 1–8 d after vaccination; the highest proportion was found on the second day after vaccination, and then the proportion gradually decreased with time. HBsAg, Hepatitis B surface antigen.

Among the 1417 neonates for whom HBsAg was measured quantitatively after receiving the hepatitis B vaccine, HBsAg positivity was mainly concentrated at 8 d after inoculation and was negative 9 d after vaccination (**Table 3**). The proportion of newborns positive for HBsAg was highest on the 2nd day after inoculation, at 42.4%, and then decreased gradually with time. The mean HBsAg-positive concentration varied with time on the days between vaccination and blood collection. It reached 0.222 (0.110–0.405) IU/mL on the 2nd day after the injection of the hepatitis B vaccine and then gradually decreased with time, and the average HBsAg-positive concentration was <0.05 IU/mL on the 9th day after vaccination (**Figure 3**).

**Table 3.** HBsAg distribution at different blood collection times after 1417 neonates were vaccinated with hepatitis B vaccine

**Interval (days)**	**Number of newborns**	**Number of newborns (%)**		**HBsAg (IU/mL) mean (range)**

		**HBsAg ≤0.06 IU/mL**	**HBsAg >0.06 IU/mL**	
1	375	283 (75.47)	92 (24.53)	0.188 (0.106–0.312)
2	205	118 (57.56)	87 (42.44)	0.222 (0.110–0.405)
3	207	145 (70.05)	62 (29.95)	0.178 (0.099–0.346)
4	147	109 (74.15)	38 (25.85)	0.209 (0.123–0.338)
5	91	76 (83.52)	15 (16.48)	0.117 (0.095–0.188)
6	55	46 (83.64)	9 (16.36)	0.088 (0.083–0.120)
7	45	43 (95.56)	2 (4.44)	–
8	29	28 (96.55)	1 (3.45)	–
9–28	263	263	0	<0.05
Total	1417	1111	306	0.186 (0.104–0.339)

Anti-HBs, hepatitis B surface antibodies; HBsAg, hepatitis B surface antigen.

### Effect of hepatitis B vaccination on the detection of serum surface antigens in vitro

**Figure 4.**
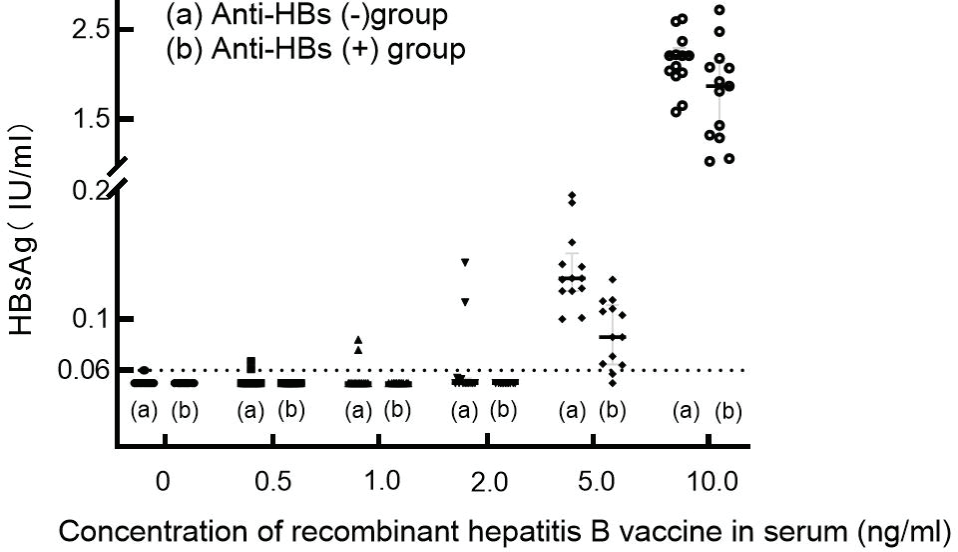
(A) HBsAg status after various concentrations of recombinant hepatitis B vaccine were added to anti-HBs (−) serum. (B) HBsAg results after various concentrations of recombinant hepatitis B vaccine were added to anti-HBs (+) serum. When the recombinant hepatitis B vaccine was not added to the serum, the HBsAg levels in both groups were ≤0.06 IU/mL. When the concentration of vaccine in serum was 0.5 ng/mL, 1 ng/mL, or 2 ng/mL, only 2 instances were HBsAg positive in the anti-HBs-negative group. When the concentration of vaccine in serum was 5 ng/mL, the HBsAg status in the anti-HBs negative group was all positive, while the proportion of HBsAg positives in the anti-HBs-positive group was 84.6%, and the average concentration of HBsAg in the anti-HBs negative group was higher than that in the anti-HBs-positive group. When the concentration of vaccine in serum was 10 ng/mL, the HBsAg status in the 2 groups was positive. Similarly, the average concentration of HBsAg in the anti-HBs negative group was higher than that in the anti-HBs-positive group. Anti-HBs, hepatitis B surface antibodies; HBsAg, hepatitis B surface antigen.

In the anti-HBs negative sample group, positive HBsAg was detected when the recombinant hepatitis B vaccine concentration in the serum was 0.5 ng/mL, the HBsAg positive was 15.4% (2/13), and the average HBsAg-positive concentration was 0.064 IU/mL. When the concentration of recombinant hepatitis B vaccine was 5 ng/mL, all samples were positive for HBsAg, and the median HBsAg concentration was a mean of 0.132 (range: 0.122–0.143) IU/mL. However, in the anti-HBs-positive sample group, HBsAg positivity was not observed until the concentration of recombinant hepatitis B vaccine was 5 ng/mL, the positive proportion was 84.6% (11/13), and the median HBsAg concentration was a mean of 0.103 (0.079–0.111) IU/mL (**Table 4**; *P* < 0.05 was considered significant). When the serum concentration of the recombinant hepatitis B vaccine was 5 ng/mL and 10 ng/mL (*P* < 0.05), there was a significant difference in the distribution of HBsAg results between the 2 groups. The HBsAg concentration was detected after different concentrations of the hepatitis B vaccine were added to the serum samples of the 2 groups, as shown in **Figure 4**.

**Table 4.** HBsAg after the recombinant hepatitis B vaccine was added to the serum samples

**Vaccine (ng/mL)**	**Group**			

	**Anti-HBs (−)**		**Anti-HBs (+)**	

	**Positive number (proportion)**	**HBsAg (IU/mL) mean (range)**	**Positive number (proportion)**	**HBsAg (IU/mL) mean (range)**
0.5	2 (15.4%)	NA	0 (0)	NA
1	2 (15.4%)	NA	0 (0)	NA
2	2 (15.4%)	NA	0 (0)	NA
5	13 (100%)	0.132 (0.122–0.143)***	11 (84.6%)	0.103 (0.079–0.111)
10	13 (100%)	2.210 (2.02–2.22)*	13 (100%)	1.87 (1.32–2.08)

* *P* < 0.05, compared with HBsAg status of the anti-HBs (+) group;

*** *P* < 0.01, compared with HBsAg status of the anti-HBs (+) group.

Anti-HBs, hepatitis B surface antibodies; HBsAg, hepatitis B surface antigen; NA, not applicable.

**Figure 5.**
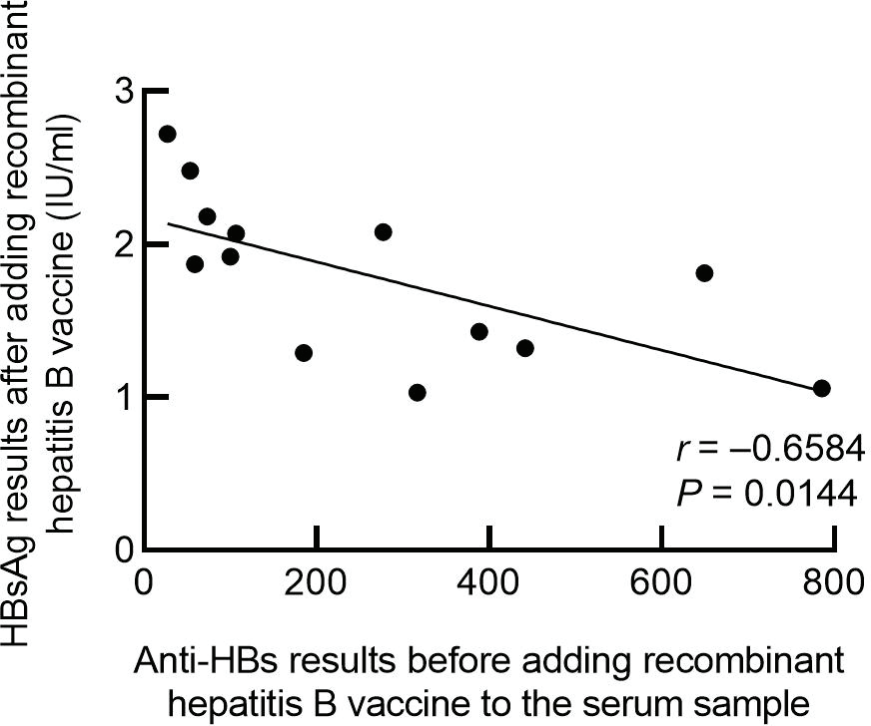
In the anti-HBs (+) group, the correlation between HBsAg and anti-HBs was analyzed when the concentration of recombinant hepatitis B vaccine was 10 ng/mL. Pearson correlation analysis was performed on the anti-HBs detection results in the serum without recombinant hepatitis B vaccine and the HBsAg detection results after the addition of the hepatitis B vaccine. The Pearson correlation, *r* = −0.658, *P* < 0.014, indicates that the original anti-HBs concentration in the serum is negatively correlated with the HBsAg levels after adding the same amount of vaccine. Anti-HBs, hepatitis B surface antibodies; HBsAg, hepatitis B surface antigen.

When the serum concentration of the recombinant hepatitis B vaccine was 10 ng/mL, all HBsAg results in the anti-HBs (+) group were positive. The anti-HBs results in the samples gave a Pearson correlation coefficient of *r* = −0.658, *P* = 0.014, indicating that the concentration of original anti-HBS in plasma was negatively correlated with the concentration of HBsAg after adding the same amount of hepatitis B vaccine (**Figure 5**).

## Discussion

A reduction in HBV infection in infants is of great importance in controlling HBV transmission. We found that the detection of HBsAg-positive newborns after vaccination is not related to HBV infection, but has a strong association with recombinant hepatitis B vaccination. However, the question of why the proportion of HBsAg-positive neonates is lower for those born to mothers who are HBsAg-positive remains. The reason may be that newborns in this group were vaccinated with hepatitis B immunoglobulin at the same time. This immunoglobulin is a highly effective exogenous antibody against hepatitis B and can react with circulating antigen, thereby affecting the detection of HBsAg [22, 23]. Because the anti-HBs in the mother are mostly IgG type and can be transmitted to the newborn through the placental circulation, this also explains the inverse relationship in **Figure 2**; the higher the concentration of anti-HBs in the mother, the lower the positive rate of HBsAg in the newborn. Nonetheless, if the maternal antenatal concentration of anti-HBs before delivery is higher, is the protective effect on neonates stronger? Hu et al. [24] have demonstrated that anti-HBs obtained by passive immunity through placental circulation in newborns may somehow weaken the antibody response to the hepatitis B vaccine. Whereas, Wang et al. [25] demonstrated that maternal anti-HBs, even at high concentrations in infants, do not inhibit the long-term immunogenicity of the hepatitis B vaccine. Although a certain amount of anti-HBs can be obtained by transplacental circulation, which can reduce HBsAg positivity after vaccination, at least to a certain extent, the effect of maternal antibody level on the immune effect after vaccination needs further examination.

To determine the starting time and duration of HBsAg-positive detection after receiving recombinant hepatitis B vaccine (derived from CHO cells), 1417 newborns analyzed for HBsAg and anti-HBs showed that HBsAg positivity mainly peaked within 8 d after inoculation and that the proportion of positive neonates was highest on the second day after vaccination. This is consistent with the duration of HBsAg positivity and the occurrence of the highest positive proportion found in other relevant studies, but not completely consistent with the peak HBsAg concentration [26,27,28]. The reasons for the inconsistency could be as follows: (1) In the present study, a domestic recombinant hepatitis B vaccine (derived from CHO cells) was selected for inoculation, and its metabolism in vivo has not been reported in the relevant literature. (2) In the present study, electrochemical luminescence was selected to detect the HBsAg quantitatively. The reagent used in this method labels the antigen directly with ruthenium tripyridinium, which is stable in binding, is not affected by the physical and chemical properties of the label, and has higher sensitivity than other quantitative methods. (3) After grouping the collected sample data, the number of samples in each group varied greatly, which may lead the result to deviate from that expected.

A serum verification experiment in vitro confirmed that the injection of the recombinant hepatitis B vaccine (derived from CHO cells) would indeed make the HBsAg quantitative test positive and confirmed that the number of maternal anti-HBs will have an impact on the newborn HBsAg detection results. However, because this experiment was performed in vitro, it could not verify the changes in the hepatitis B vaccine in different stages, such as diffusion and absorption, or the involvement in immune response metabolism.

## Conclusion

We suggest that when the domestic recombinant hepatitis B vaccine (derived from CHO cells) is used for vaccination, HBsAg detection should preferably be conducted at least 1 week after inoculation, and the family members should be informed of the possible reasons when the report is interpreted to avoid unnecessary anxiety and the loss of further examination.

Nevertheless, laboratory technicians should be vigilant for neonatal early HBsAg-positive results. Suspected hepatitis B infection should be eliminated by neutralization experiments, a hepatitis B nucleic acid test, and other methods, combined with the mother’s history of hepatitis B and hepatitis B quantitative results for comprehensive analysis and judgment.
